# Factors Influencing Consumer Willingness to Use AI-Driven Autonomous Taxis

**DOI:** 10.3390/bs14121216

**Published:** 2024-12-18

**Authors:** Tingyu Liu, Yizhou Zhang, Mengze Zhang, Min Chen, Shangchun Yu

**Affiliations:** 1 Department of Economics, Sejong University, Seoul 05006, Republic of Korea; 23150512@sju.ac.kr (T.L.); chenm2309@163.com (M.C.); yushang2020@gmail.com (S.Y.); 2 Economic and Trade Department, Yancheng Polytechnic College, Yancheng 224005, China; 3 Department of Global Business, Anyang University, Anyang-si 14028, Republic of Korea; alisonzhang27@gmail.com

**Keywords:** autonomous taxis, intention to use, perceived risk, Theory of Planned Behavior (TPB), Technology Acceptance Model (TAM)

## Abstract

The advancement of autonomous driving technology, particularly Tesla’s launch of its new Robotaxi, marks a transformation in transportation. Understanding the theoretical mechanisms that drive consumers’ intention to use autonomous taxis is essential. This study develops a structural equation model (SEM), extending the applicability of the TAM and TPB model, and incorporates external factors like attitudes, subjective norms, traffic efficiency, and perceived cost–benefit into the model to analyze their impact on consumers’ perceived characteristics (perceived usefulness and perceived ease of use). A survey of 427 valid responses revealed that attitudes, subjective norms, and perceived cost–benefit all have significant positive impacts on perceived usefulness and ease of use, which, in turn, are the primary drivers of consumers’ intention to use. Additionally, perceived risk significantly weakens the positive effects of perceived usefulness and ease of use on the intention to use, underscoring its critical moderating role in the technology acceptance process. This paper suggests strategies to enhance consumer acceptance, including strengthening user perception through marketing and public experience activities, optimizing technology to improve user experience, reinforcing safety and privacy measures to reduce perceived risk, and highlighting the insurance mechanism, convenience, and economic benefits of autonomous taxis in marketing.

## 1. Introduction

AI (artificial intelligence) has emerged as the core driving force behind a new wave of industrial transformation, profoundly impacting the global economy, labor productivity, healthcare, smart cities, and transportation [1]. The development of deep learning and large language models (LLMs) has positioned AI as a critical tool across various industries. A representative example is the widespread application of AI technologies like ChatGPT, showcasing their potential in automation and natural language processing [2]. Tesla has unveiled its new Robotaxi, a driverless vehicle without a steering wheel or pedals, marking a new phase in autonomous driving technology and signaling a revolutionary transformation in transportation.

Autonomous vehicles (AVs) are gradually entering the market, offering consumers a new user experience [3]. In recent years, tech companies like Waymo, Tesla, and Baidu Apollo have ramped up their research and development in autonomous driving technology, aiming to transform the traditional taxi industry and enhance transportation efficiency. These advancements are expected to profoundly impact urban transportation, environmental protection, and consumer travel [4,5,6]. In China, Baidu Apollo Go represents a significant advancement in applying autonomous driving technology to real-world scenarios. In 2024, Baidu Apollo Go deployed 400 autonomous vehicles in Wuhan, completing over 6 million trips. Users can access Apollo Go through Baidu Maps or the “Apollo Go” app. The booking process is straightforward: open Baidu Maps, select “Taxi,” then “Autonomous Driving.” Choose the pick-up and drop-off points, as well as the number of passengers (up to two), then confirm the booking by entering the passenger’s name and ID. The driver’s seat has two displays showing the vehicle’s driving status, including lane markings and high-definition maps, while passengers can view trip details on a screen in front of the rear seats. Apollo Go has successfully navigated Wuhan’s diverse road conditions, covering complex urban scenarios citywide and offering residents a convenient, driverless travel experience.

Despite the significant attention given to autonomous taxi technology and its market deployment, considerable uncertainty remains about consumer acceptance of autonomous driving. Studies suggest that consumer acceptance of autonomous driving is influenced by technological, economic, and ethical factors. Research shows that optimal route planning, improved traffic efficiency, and ensuring safety are core challenges for autonomous taxis in different scenarios [7,8,9]. While autonomous taxis offer significant social and environmental benefits [10], ethical dilemmas involving traffic accidents and liability could shape public perceptions [11]. Consumers also express concerns regarding the safety and privacy of autonomous vehicles. Studies show that safety concerns often impact usage intention more than cost factors [10,12]. As AI technology transforms vehicles into more intelligent, computerized systems, privacy issues are becoming increasingly prominent. Perceptions of privacy risks directly affect users’ acceptance of autonomous vehicles [13,14]. As autonomous taxi technology becomes commercialized, understanding consumer behavior and the intention to use is increasingly important.

Accordingly, this research seeks to explore the primary factors affecting consumers’ willingness to use autonomous taxis. Specifically, it examines core variables like perceived usefulness, ease of use, and intention to use, as well as the relationships between external factors (e.g., attitudes, subjective norms, perceived traffic efficiency, and perceived cost-benefit) and the core variables. These analyses aim to provide a theoretical foundation for the commercialization of autonomous vehicles. A deeper understanding of consumers’ intentions and influencing factors can help companies optimize technology and services while supporting policymakers in developing effective market strategies.

This study integrates the Theory of Planned Behavior (TPB) and the Technology Acceptance Model (TAM) to comprehensively analyze consumer behavior. TPB explains how individuals’ attitudes, subjective norms, and perceived behavioral control influence their choices [15], making it suitable for autonomous driving scenarios. TAM, conversely, reveals how perceived usefulness and perceived ease of use shape consumers’ attitudes toward adopting new technologies [16]. This research incorporates external variables, such as perceived traffic efficiency and cost-benefit, into the model while introducing perceived risk as a moderating variable, extending the application of TAM and TPB to the field of autonomous driving. It provides a novel perspective on user behavior analysis by examining the impact of these factors on consumers’ intention to adopt autonomous taxis and offering practical recommendations for industry stakeholders. The findings aim to guide companies in optimizing technology design and user experience, thereby enhancing their market competitiveness.

The structure of this paper is as follows: Initially, the introduction and research background are presented. Next, the study’s conceptual framework and model hypotheses are presented. A quantitative analysis is performed using key data on consumers’ intentions to use autonomous taxis. Subsequently, the empirical analysis and discussion of results are presented, focusing on commonalities in both theoretical and practical aspects. Finally, the study’s limitations are discussed, and recommendations for future research are provided.

## 2. Literature Review and Hypothesis

### 2.1. Theory of Planned Behavior (TPB)

The Theory of Planned Behavior (TPB) is a psychological model that explains and predicts individual behavior [15]. In the fields of autonomous driving and AI, new research has revealed that the key components of the Theory of Planned Behavior (TPB)—attitudes, subjective norms, and perceived behavioral control—play a significant role in promoting acceptance of artificial intelligence and autonomous vehicles. These findings highlight the central position of TPB in understanding the adoption behaviors of new technologies [17,18,19].

Building on this, scholars have employed the Extended Theory of Planned Behavior (TPB) to deeply investigate the influence of normative factors on the adoption intention of autonomous vehicles (AVs) and the formation mechanism of potential users’ willingness to use them [20,21]. The findings indicate that attitudes and subjective norms have a significant positive impact on adoption intention. Moreover, a study on the acceptance of autonomous vehicles among the Malaysian public further validates the pivotal psychological driving role of attitudes and subjective norms in technology adoption [22]. This study integrates attitudes and subjective norms from TPB into the external variables of the Technology Acceptance Model (TAM) to better explain consumers’ intention to use autonomous taxis.

### 2.2. Technology Acceptance Model (TAM)

The core concept of the Technology Acceptance Model (TAM) is to explain the behavioral mechanisms behind users’ adoption and utilization of new technology [16]. Studies based on the Unified Theory of Acceptance and Use of Technology (UTAUT) incorporate factors such as performance expectancy, effort expectancy, social influence, and facilitating conditions, offering a broader analytical perspective [22,23]. Research indicates that the UTAUT model is more applicable in contexts involving perceived anthropomorphism, while the TAM is better suited for individual user-centered analyses [23,24,25].

To further enrich the theoretical framework, researchers have expanded the Technology Acceptance Model (TAM), particularly focusing on the critical roles of perceived ease of use and perceived usefulness in the adoption of emerging technologies. Studies have demonstrated that these factors significantly influence user acceptance and utilization behavior of technologies such as AI robots and autonomous vehicles [26,27]. Perceived ease of use, as an indicator of how straightforward it is to operate the technology, and perceived usefulness, as a measure of whether the technology enhances efficiency in work or daily life, have been proven to be decisive factors in consumer adoption of new technologies. These two core variables not only shape consumers’ attitudes toward new technologies but also directly drive their usage decisions [23,28].

Scholars have applied the TAM to study the associations between perceived usefulness, ease of use, and the acceptance of autonomous vehicles (AVs) from the perspective of AV information systems [29], exploring consumer acceptance of autonomous vehicles (AVs) from multiple perspectives. Studies have shown that dispositional mindfulness significantly enhances users’ acceptance of autonomous vehicles (AVs), with such individuals more likely to become early adopters. Their acceptance intentions are further strengthened through improved perceived usefulness [30]. Additionally, the TAM serves as a primary framework for explaining AV acceptance behaviors, particularly emphasizing the critical roles of risk perception and functional benefits in user decision-making [31]. Trust has also been identified as a key factor influencing AV adoption in developing countries, significantly enhancing usage intentions by increasing perceived ease of use and reducing risk perception [32].

Research on autonomous driving technology in the field of transportation indicates that the advantages of autonomous driving, such as improved accessibility, safety, traffic management, reduced emissions, and enhanced comfort, have encouraged more consumers to adopt this technology [33]. Additionally, autonomous driving technology significantly reduces travel costs, enhancing consumers’ perception of cost–benefit. Research indicates that perceived cost–benefit crucially influences consumer preferences, with lower travel costs increasing acceptance of autonomous driving technology [34,35,36,37].

Therefore, this paper incorporates attitudes, subjective norms, perceived traffic efficiency, and perceived cost–benefit as external variables in the TAM, thoroughly exploring the impact of these factors on consumers’ intention to adopt autonomous taxis. Additionally, this study integrates key elements from both the Technology Acceptance Model (TAM) and the Theory of Planned Behavior (TPB) to provide a more systematic explanation of consumers’ acceptance and usage of autonomous driving technology.

### 2.3. Research Hypotheses

To guide the research, this study builds on the Technology Acceptance Model (TAM) and integrates consumers’ perceived characteristics of autonomous taxis. Thirteen hypotheses regarding the relationships among various variables are proposed and summarized below.

#### 2.3.1. External Variables and the Technology Acceptance Model (TAM)

This study categorizes external variables into four dimensions: attitudes, subjective norms, perceived traffic efficiency, and perceived cost–benefit. The two core concepts of the Technology Acceptance Model (TAM), namely “perceived ease of use” and “perceived usefulness,” are influenced by these external variables. Attitude, a key factor in TPB, has been shown to positively influence consumers’ views on autonomous driving, especially regarding environmental benefits, driverless travel feasibility, and reduced driver fatigue [37,38]. Autonomous driving also adheres well to traffic regulations [39] and enhances work and life efficiency, indicating high usefulness. Studies suggest that consumers generally find autonomous driving easy to operate and control [40].

Subjective norms are a key construct in TPB, and research shows they are crucial in fostering public acceptance of autonomous vehicles [41]. As autonomous vehicles are expected to become a main mode of transportation [19], subjective norms and perceived behavioral control directly impact public acceptance of autonomous driving technology [42]. Studies also show that subjective norms significantly influence the intention to use autonomous vehicles, as individuals are affected by the expectations and social pressure of others when deciding to use autonomous vehicles [43]. This suggests that subjective norms play an important role in enhancing the perceived usefulness and ease of use of autonomous taxis.

Autonomous vehicles (AVs) significantly impact the safety and efficiency of transportation networks. Research shows that AV technology enhances traffic efficiency and safety by optimizing routes, reducing human errors, and improving automated control [44]. Additionally, AVs improve vehicle operation and significantly reduce energy consumption and environmental impact in transportation. AV usage patterns can be optimized based on consumer preferences, further increasing the technology’s utility [45]. AVs have the potential to transform transportation systems, offering unprecedented efficiency, safety, mobility, and improved traffic flow while enhancing affordability and convenience [46,47]. As a result, perceived improvements in traffic efficiency—such as reduced congestion, shorter travel times, and enhanced travel experiences—significantly influence consumers’ perception of the usefulness of autonomous vehicles. When users recognize these benefits, they are more inclined to perceive AVs as beneficial for their daily lives.

Autonomous vehicles can improve traffic flow, reduce accidents, mitigate social exclusion, and enhance travel time efficiency [48]. Potential users’ initial trust is mainly reflected in their perception of the cost–benefit of autonomous taxis, especially regarding economic factors and travel efficiency. This makes them more willing to accept the technology [49], increasing its perceived usefulness. If autonomous taxis have reasonable pricing and simple operation, consumers will perceive the technology as more convenient, enhancing their intention to use it. Based on the above research, the following hypotheses are proposed:

**H1a.**  *Consumers’ positive attitudes toward autonomous taxis positively impact perceived ease of use.*

**H1b.**  *Consumers’ positive attitudes toward autonomous taxis positively impact perceived usefulness.*

**H2a.**  *Subjective norms positively impact perceived ease of use.*

**H2b.**  *Subjective norms positively impact perceived usefulness.*

**H3a.**  *The perceived traffic efficiency of autonomous taxis positively impacts perceived ease of use.*

**H3b.**  *The perceived traffic efficiency of autonomous taxis positively impacts perceived usefulness.*

**H4a.**  *The perceived cost–benefit of autonomous taxis positively impacts perceived ease of use.*

**H4b.**  *The perceived cost–benefit of autonomous taxis positively impacts perceived usefulness.*

#### 2.3.2. Technology Acceptance Model and Consumer Intention to Use

Various factors significantly influence behavioral intention (BI) in technology adoption. Studies highlight the importance of trust, algorithm transparency, and ethical considerations in shaping behavioral intentions. For example, the interaction between human–AI collaboration types (AI-dominant vs. AI-assisted) and outcome expectations significantly affects adoption intentions, with algorithm transparency mitigating the negative impact of adverse expectations [50]. Additionally, IT mindfulness enhances perceived benefits, thereby increasing consumers’ intention to adopt autonomous driving technologies [51].

Ethical algorithms in autonomous driving significantly impact users’ trust and adoption intentions in critical situations. Similarly, studies on virtual human technologies demonstrate that optimizing transparency and ethical standards can effectively enhance user trust and adoption intentions [52,53]. Privacy risks and data security issues are also critical barriers to technology acceptance. Research indicates that reducing privacy risks and perceived defects through word-of-mouth effects not only improves recommendation intentions but also indirectly promotes technology adoption [54,55].

Building on these various factors, the Technology Acceptance Model (TAM) further emphasizes the central role of perceived ease of use and perceived usefulness in consumer’s intention to use. Studies confirm a positive relationship between perceived ease of use and users’ attitudes toward technology [56], and the effect of ease of use on usefulness is also empirically supported [57]. Users are more likely to adopt new technologies when they offer more advantages and convenience compared to existing ones [58]. Additionally, the stronger the perception of ease of use, the more positive users’ attitudes toward the technology, which enhances their intention to use it [16]. Research shows that when consumers use AI chatbots, ease of use positively influences usefulness [59]. Studies also find that university students are more likely to adopt ChatGPT when they perceive it as useful and easy to use [60]. Based on this, the following hypotheses are proposed:

**H5.**  *Perceived ease of use has a significant positive impact on perceived usefulness.*

**H6.**  *Perceived ease of use positively impacts consumers’ intention to use autonomous taxis.*

**H7.**  *Perceived usefulness positively impacts consumers’ intention to use autonomous taxis.*

#### 2.3.3. The Moderating Role of Perceived Risk

Perceived risk refers to the anticipation of potential losses. The higher the perceived likelihood of loss, the greater the perceived risk [61]. It is defined as consumers’ negative evaluation of the unpredictability and uncertainty of product outcomes [62]. The public is particularly concerned about the safety and privacy risks of autonomous vehicles, which are seen as the two most important issues [63]. Research suggests that increasing perceived usefulness (PU) and reducing perceived risks of autonomous vehicles (AVs) can enhance consumers’ trust, promoting their usage [64]. Additionally, studies demonstrate that perceived risk significantly affects the intention to use, with perceptions of safety strongly impacting trust in autonomous driving [65]. Higher perceived safety leads to greater trust in autonomous vehicles [66]. Based on this, the following hypotheses are proposed:

**H8a.**  *Perceived risk negatively moderates the relationship between perceived usefulness and intention to use.*

**H8b.**  *Perceived risk negatively moderates the relationship between perceived ease of use and intention to use.*

**Figure 1:**
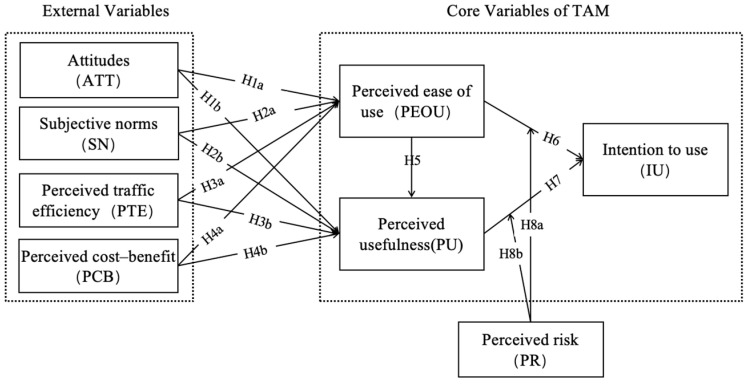
Research model.

Based on the above literature review, we developed our research model, as presented in Figure 1.

## 3. Materials and Methods

### 3.1. Measurement Scales and Instruments

The measurement items in the research model are adapted from the existing literature. This approach ensures validity and reliability, as these items have been validated in previous research with robust theoretical foundations and practical relevance. This approach ensures validity and reliability, as these items have been validated in previous studies with strong theoretical support and practical applicability. Using measurement items from the established literature also maintains consistency with other studies, facilitates cross-study comparisons, and enhances the credibility and rigor of the research. The specific items are listed in Table 1. In the TAM, external variables are divided into four dimensions: attitudes, subjective norms, perceived traffic efficiency, and perceived cost–benefit. The measurement items for attitudes are adapted from Arachchi and Samarasinghe (2023) and Kerschner and Ehler (2016) [67,68]; for subjective norms from Duong (2024) and Taylor and Todd (1995) [69,70]; for perceived traffic efficiency from Narayanan et al. (2022) and Wang et al. (2024) [71,72]; and for perceived cost–benefit from Kiviniemi and Duangdao (2009) [73]. Furthermore, the core TAM elements—perceived usefulness and perceived ease of use—are derived from Davis (1989), Majrashi (2022), Arachchi and Samarasinghe (2023), and Pillai et al. (2020) [16,67,74,75], respectively. The measurement items for perceived risk are adapted from Chopdar et al. (2018) and Ma et al. (2022) [76,77]. Finally, the measurement items for intention to use are adapted from Lin et al. (2020) [78].

The questionnaire items used in this study were originally in English. To ensure the translated questionnaire aligns with the Chinese context, we adopted the “forward-backward translation” method. First, bilingual experts translated the English questionnaire into Chinese. Then, independent bilingual experts back-translated the Chinese version into English to ensure accuracy and consistency. To further improve accuracy, experts in the field reviewed and revised the translation, ensuring the questionnaire meets academic standards and is easily understood by Chinese respondents. All items were assessed on a seven-point Likert scale, from “strongly disagree” (1) to “strongly agree” (7).

The questionnaire is composed of two sections: the first part collects respondents’ basic demographic information, while the second part focuses on behavioral research, specifically examining factors influencing consumers’ intention to use.

### 3.2. Data Collection

Data for this study were gathered via an online survey from August to September 2024, targeting respondents from Wuhan, Hubei Province, where the “Apollo Go” service is available. To ensure sample relevance, the first question asked whether respondents had used the “Apollo Go” service at least once in the past month. Respondents who answered “yes” continued with the survey, while those who answered “no” were automatically excluded. This ensured that all participants were actual users, enhancing data relevance and accuracy. The survey was anonymous, and participants were informed that all data would be utilized exclusively for academic research purposes, with personal information kept confidential. Respondents were also assured that there were no correct or incorrect answers, and the aim was to capture their true thoughts and experiences.

A total of 480 questionnaires were collected in this survey, exceeding the recommended minimum sample size for each research item (i.e., the sample size should be at least ten times the number of items, as suggested by Costello and Osborne, 2005 [79]). A power analysis was conducted using SPSSAU with a significance level (α) of 0.05, a power value (1 − β) of 0.8, and a correlation coefficient of 0.4, indicating a minimum required sample size of 395. The actual sample size in this study meets this requirement, ensuring the statistical power and reliability of the structural equation model’s path analysis. Z-scores were calculated for each respondent, and scores exceeding ± 3 were considered outliers and excluded [80]. This resulted in 427 valid questionnaires, achieving an effective response rate of 88.96%.

### 3.3. Common Method Bias

Following Podsakoff et al. (2003), we implemented procedural controls to minimize potential common method bias. We ensured respondent anonymity and emphasized that there were no right or wrong answers. Additionally, the order of questionnaire items was randomized so that each respondent saw a different sequence. These procedural controls help reduce common method bias [81].

Statistical controls were applied to the data. Harman’s single-factor test examined potential common method bias. We utilized AMOS 26.0 to construct a single-factor structural equation model and examined its fit. According to CFA testing criteria, if the fit indices of the single-factor model fail to meet the recommended thresholds, it indicates that common method bias is not severe [82]. The fit indices of the single-factor model were as follows: χ^2^ = 2503.542, df = 324, χ^2^/df = 7.732 (>3), SRMR = 0.075 (>0.05), RMSEA = 0.126 (>0.08), GFI = 0.649 (<0.9), IFI = 0.733 (<0.9), CFI = 0.732 (<0.9), and TLI = 0.710 (<0.9), all of which did not reach the recommended fit standards. Thus, common method bias is within an acceptable range and is unlikely to affect subsequent statistical analyses [82].

## 4. Data Analysis and Results

### 4.1. Sample Characteristics

The gender distribution includes 218 male respondents (51.05%) and 209 female respondents (48.95%), aligning with the natural gender balance. The largest age group is 20–29 years old, representing 70.3% of the sample. Most respondents hold a bachelor’s degree (55.5%). The largest occupational group is professional and technical personnel, comprising 175 respondents (40.98%). The highest income group is under CNY 3000, reported by 176 respondents. The most frequent usage in the past month was 3–5 times, reported by 177 respondents (Table 2).

### 4.2. Measurement Model

Before analyzing the questionnaire data, it is essential to assess the reliability and validity of the measurement instruments. Reliability ensures consistent measurement results, while validity confirms that the tools measure what they are intended to. In this study, confirmatory factor analysis (CFA) was used along with Cronbach’s α, composite reliability (CR), and average variance extracted (AVE) to assess internal consistency. According to established criteria, Cronbach’s α and CR should exceed 0.7, and AVE should be greater than 0.5 [80,83].

Table 3 presents the results of the reliability and validity analysis. All CR values exceed the 0.7 threshold, indicating good reliability. Similarly, all AVE values are above 0.6. All Cronbach’s α coefficients are greater than 0.7, confirming high internal consistency. These results demonstrate strong reliability and validity, making the data suitable for further analysis.

The results of the model fit assessment for the measurement model are shown in Table 4. The model demonstrates an appropriate fit to the data using maximum likelihood estimation, with all indicators meeting recommended thresholds [80]. The model fit indices are as follows: χ^2^/df = 1.771 (<3), SRMR = 0.028 (<0.08), RMSEA = 0.043 (<0.08), GFI = 0.919 (>0.9), IFI = 0.972 (>0.9), CFI = 0.972 (>0.9), and TLI = 0.967 (>0.9). All indices meet the required standards, indicating a good model fit.

### 4.3. Discriminant Validity

To confirm each factor’s unique explanatory power, the square root of the AVE should exceed the correlation coefficients between that factor and others. The results in Table 5 confirm that this requirement is met, indicating good discriminant validity [84].

### 4.4. Structural Model

This study used maximum likelihood estimation with AMOS 26.0 to assess the structural model fit. All fit indices were required to meet standard thresholds [80]. The structural model fit indices are as follows: χ^2^/df = 1.791 (<3), SRMR = 0.028, RMSEA = 0.04 (<0.08), GFI = 0.926 (>0.9), IFI = 0.975 (>0.9), CFI = 0.975 (>0.9), and TLI = 0.970 (>0.9). As shown in Table 6, all fit indices meet the required standards, indicating a good fit with the data.

**Figure 2:**
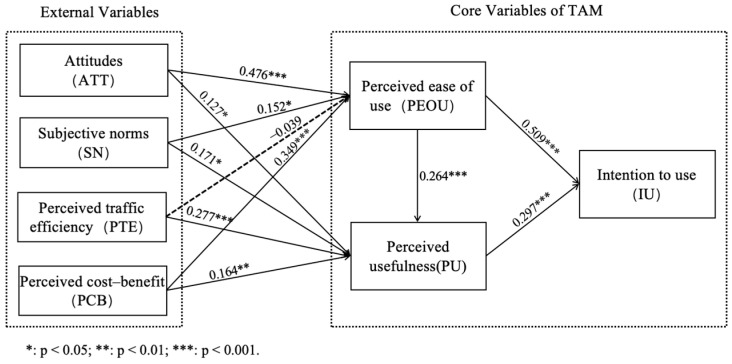
The results of path analysis.

As shown in Table 7, attitude (ATT), subjective norms (SN), and perceived cost–benefit (PCB) all have significant positive effects on perceived ease of use (PEOU) and perceived usefulness (PU), supporting hypotheses H1a, H2a, H4a, H1b, H2b, and H4b. Perceived traffic efficiency (PTE) does not significantly affect perceived ease of use, leading to the rejection of H3a, but it has a significantly positive impact on perceived usefulness, supporting H3b. Additionally, perceived ease of use significantly influences both perceived usefulness and intention to use, supporting H5 and H6. Perceived usefulness also has a significant impact on the intention to use, supporting H7. Figure 2 depicts the result of the path analysis.

### 4.5. Moderation Analysis

PROCESS v.3.5 was used to analyze the moderating effects. PROCESS is a statistical software tool used in SPSS and SAS. It is widely used in social sciences research to perform complex path analyses, mediation, moderation, and conditional process analyses [85,86]. As shown in Table 8, Model 1 indicates that perceived ease of use (PEOU) has a statistically significant positive impact on intention to use (IU) (coefficient = 0.450, *p* < 0.001), while perceived risk (PR) has a significant negative effect (coefficient = −0.283, *p* < 0.001). The interaction between PEOU and PR also has a significant negative effect on the intention to use (coefficient = −0.129, *p* < 0.001), indicating that perceived risk significantly moderates the effect of PEOU on IU. Model 1 explains 44.1% of the variance (R^2^ = 0.441), supporting hypothesis H8a.

In Model 2, perceived usefulness (PU) has a statistically significant positive impact on intention to use (IU) (coefficient = 0.416, *p* < 0.001), while perceived risk (PR) maintains a statistically significant negative impact (coefficient = −0.317, *p* < 0.001). The interaction between PU and PR significantly moderates the intention to use with a negative effect (coefficient = −0.080, *p* < 0.01). Model 2 explains 39.6% of the variance (R^2^ = 0.396), supporting hypothesis H8b. Overall, both perceived ease of use and perceived usefulness significantly influence intention to use, with perceived risk moderating and weakening their positive effects.

## 5. Conclusions and Discussion

### 5.1. Principal Findings

This study aimed to investigate the primary factors affecting consumers’ willingness to use autonomous taxis. An empirical analysis of attitudes (ATT), subjective norms (SN), perceived traffic efficiency (PTE), perceived cost–benefit (PCB), perceived ease of use (PEOU), and perceived usefulness (PU) validated most of the proposed hypotheses. The findings highlight the significant role these factors play in shaping consumers’ intention to use autonomous taxis.

The results show that attitude, subjective norms, and perceived cost–benefit have significant positive impacts on both perceived ease of use and perceived usefulness, supporting H1a, H2a, H4a, H1b, H2b, and H4b. Consumers’ positive attitudes, subjective norms, and perceptions of cost–benefit significantly enhance their views of the technology’s ease of use and usefulness. This aligns with Ajzen’s (1985) Theory of Planned Behavior (TPB) [15] and Davis’s (1989) Technology Acceptance Model (TAM) [16], showing that external factors are crucial in consumers’ acceptance of new technologies. The study further reveals that attitude, subjective norms, and perceived behavioral control positively influence perceived usefulness and ease of use, which indirectly impact users’ intention to adopt Shared Autonomous Vehicles (SAVs). This finding underscores the essential role of user attitude, social norms, and perceived utility in the technology adoption process [87,88]. To promote widespread acceptance, marketing campaigns and public experience activities can familiarize users with the advantages of autonomous taxis, fostering positive attitudes. Industry experts and policy guidance can create a positive social atmosphere, leveraging subjective norms while highlighting the economic advantages of autonomous taxis can enhance perceptions of cost–benefit and drive broader adoption.

However, perceived traffic efficiency (PTE) did not significantly affect perceived ease of use (H3a was rejected); however, it exhibited a significant positive effect on perceived usefulness (H3b was supported). This could be attributed to the fact that while artificial intelligence (AI) technology enhances the decision-making capabilities and overall performance of autonomous vehicles, such advancements have not directly simplified user operations [89]. Consequently, although improved traffic efficiency strengthens the vehicle’s perceived usefulness, it does not necessarily translate into enhanced ease of use.

To address this limitation, innovative user interface designs, such as the implementation of interactive displays, can significantly improve user experience by enhancing environmental awareness [90]. These designs not only directly improve system usability but also indirectly enhance the overall travel experience through more intuitive information presentation and guided interactions. Therefore, the development of autonomous vehicle technology should focus on transforming technological advancements into practical operational convenience for users, particularly in areas such as booking, payment, riding processes, and human–machine interaction, to achieve substantial improvements in ease of use.

The low transportation costs and reduced traffic congestion highlighted during the Tesla Robotaxi launch further emphasize the roles of perceived cost–benefit and traffic efficiency in shaping consumers’ perceived usefulness. Our study confirmed the positive impact of these factors on perceived usefulness, indicating that consumers pay close attention to the economic and traffic-related benefits of autonomous taxis.

Additionally, perceived ease of use significantly impacts both perceived usefulness and intention to use (supporting H5 and H6). This finding supports the research conclusion that perceived ease of use has a direct influence on behavioral intention and indirectly affects it through perceived usefulness [91,92,93]. The significant effect of perceived usefulness on intention to use was also confirmed (supporting H7). These results align with TAM, showing that ease of use, directly and indirectly, influences intention to use by enhancing perceptions of the technology’s usefulness. From the behavioral economics perspective, consumers weigh utility maximization against behavioral imitation when making decisions [94]. If users perceive autonomous taxis as easy to operate and capable of saving time and costs, their perceived utility will increase significantly, thereby enhancing their adoption intention. This perspective highlights the dual impact of ease of use and usefulness on consumer behavior, emphasizing the critical role of intuitive interface design and marketing strategies in driving the adoption of autonomous driving technologies.

The findings indicate that perceived risk (PR) significantly reduces the positive effect of perceived ease of use and perceived usefulness on the intention to use (supporting H8a and H8b). Although consumers acknowledge the practical value of autonomous vehicles (AVs), concerns about safety, data privacy breaches, and functional failures considerably weaken their intention to adopt the technology [95,96]. Among these factors, cybersecurity related to privacy emerges as the most critical determinant of consumers’ trust and acceptance of AVs, directly influencing their perception of the technology’s reliability [95]. To mitigate such concerns, integrating advanced technologies such as deep reinforcement learning (DRL) and risk prediction mechanisms can enhance AVs’ ability to respond to potential threats by combining multi-source data and real-time risk assessments, reducing accident risks and strengthening users’ sense of security [97].

Addressing privacy and safety concerns requires companies to implement robust regulatory compliance and transparent data management practices, demonstrated through publicly available testing reports and risk assessments [98]. Moreover, introducing liability insurance mechanisms can provide users with financial protection in case of accidents, alleviating concerns about potential economic losses. Policymakers should enforce privacy protection regulations by defining clear responsibilities for data management while promoting consumer oversight and industry self-regulation. Through a comprehensive strategy involving technological advancements, privacy safeguards, insurance mechanisms, and multi-stakeholder governance, companies can effectively reduce consumers’ perceived risks, enhance trust, and increase their willingness to adopt autonomous taxis, providing valuable insights for market expansion and policy development.

Perceived risk is especially important for Tesla’s Robotaxi. The design, which eliminates the steering wheel and pedals, showcases technological confidence but may also heighten consumer concerns about safety and reliability. This aligns with our findings that perceived risk significantly weakens the effect of perceived usefulness and ease of use on the intention to use. This underscores the importance of addressing these concerns in promoting and adopting autonomous vehicles like Tesla’s Robotaxi.

### 5.2. Implications

Theoretical Implications: This study integrates the TAM and the TPB model to analyze key factors influencing consumers’ willingness to use autonomous taxis. It confirms the importance of attitudes, subjective norms, perceived traffic efficiency, perceived cost–benefit, perceived ease of use, and usefulness while also revealing the moderating role of perceived risk. This expands the application of both TPB and TAM in the context of autonomous driving technology.

Practical Implications: This study proposes a series of practical strategies to promote the market adoption of autonomous taxis, focusing on technology optimization, risk management, and enhancing consumer acceptance. First, marketing and user experience initiatives play a critical role. Public testing events and social media campaigns can give consumers a more direct experience of the convenience and safety of autonomous taxis, fostering a positive attitude toward their adoption. Additionally, technology design optimization is essential for enhancing the user experience. Improving human–machine interaction interfaces, simplifying booking and payment processes, and introducing diverse ride options (such as instant booking and carpooling services) can enhance the system’s ease of use and practicality.

Enhancing safety and privacy protection mechanisms is a core strategy for reducing consumers’ perceived risks. Companies should adopt advanced security algorithms, implement robust data protection measures, and provide financial coverage through liability insurance mechanisms to alleviate concerns about privacy breaches and potential economic losses. Finally, by emphasizing practicality and convenience in marketing communications, including cost savings, reduced traffic congestion, and improved travel efficiency, companies can effectively transform technological advantages into perceived consumer value, ultimately boosting consumers’ willingness to adopt the technology. These integrated measures can significantly advance the market application of autonomous taxis and contribute to the development of a future-oriented intelligent transportation ecosystem.

### 5.3. Limitations and Directions for Future Research

While this research presents significant insights into consumer acceptance of autonomous taxis, it has several limitations. First, the data were primarily sourced from Wuhan, China. Given the differences in topography, traffic conditions, travel patterns, culture, and economic factors across cities, the applicability of the findings to other areas may be limited. Additionally, the study focused on perceived usefulness, ease of use, and risk, while other factors—such as trust, technological maturity, or social support—were not fully considered, potentially leading to incomplete coverage of influencing factors.

To address these limitations, future research could expand in the following directions. First, conducting cross-regional and cross-cultural comparative studies across different cities and regions in China would help explore how cultural values, policy environments, and market maturity influence consumers’ willingness to use autonomous taxis. Additionally, by integrating the global development trends of autonomous driving technology, future studies could investigate consumer behavior in multiple scenarios, such as shared mobility services, logistics delivery, and autonomous buses, thereby extending the existing theoretical framework.

Furthermore, incorporating a multi-stakeholder perspective (including government, businesses, and consumers), combined with technology diffusion and innovation models, would provide deeper insights into the complex mechanisms of technology adoption.

Finally, future research should analyze how different policy environments and market conditions shape the adoption paths of autonomous driving technology. This global perspective would not only expand the applicability of current theories but also offer valuable guidance for international policy development and commercial applications.

## Figures and Tables

**Table 1:** Measurement scales and items.

Latent Variable	Coding	Indicators	Items
Attitudes	ATT	ATT1	I have a positive overall attitude towards autonomous taxis.
		ATT2	I believe that autonomous taxi technology is valuable.
		ATT3	I believe that autonomous taxis can improve my travel experience.
Subjective norms	SN	SN1	The important people around me think I should use autonomous taxis.
		SN2	My friends and family expect me to try using autonomous taxis.
		SN3	In my social circle, using autonomous taxis is widely accepted.
Perceived traffic efficiency	PTE	PTE1	I believe that autonomous taxis can reduce traffic congestion caused by human driving errors.
		PTE2	The use of autonomous taxis, with their efficient computing power, can improve overall road traffic efficiency.
		PTE3	I think autonomous taxis can enhance urban traffic flow.
		PTE4	Autonomous taxis can minimize travel delays during peak hours or heavy traffic conditions.
Perceived cost– benefit	PCB	PCB1	I believe that the cost of using autonomous taxis is reasonable.
		PCB2	Using autonomous taxis can help me save both money and time.
		PCB3	I think autonomous taxis offer a high cost–performance ratio.
Perceived usefulness	PU	PU1	Using autonomous taxis improves my travel efficiency.
		PU2	Using autonomous taxis allows me to complete my travel plans more quickly.
		PU3	Using autonomous taxis makes my travel experience more relaxing.
		PU4	I believe that autonomous taxis are very useful for my travel needs.
Perceived ease of use	PEOU	PEOU1	For me, learning how to use and operate autonomous taxis is simple and effortless.
		PEOU2	It is easy to book a ride on the autonomous taxi platform, and finding my desired destination is very convenient.
		PEOU3	I can easily remember how to complete tasks on the autonomous taxi platform.
		PEOU4	Overall, I find using autonomous taxis to be simple.
Perceived risk	PR	PR1	I am concerned about the safety of autonomous taxis.
		PR2	Using autonomous taxis may lead to the leakage of my personal information.
		PR3	I believe that to reduce the risk of privacy leakage, I should follow relevant safety guidelines when using autonomous taxis.
Intention to use	IU	IU1	I am open to trying autonomous taxis in the future.
		IU2	If given the opportunity, I would prefer to choose autonomous taxis.
		IU3	I plan to use autonomous taxis for my travels in the future.

**Table 2:** Sample characteristics.

Characteristics	Number (*N* = 427)	Percentage (%)
**gender**		
Male	218	51.05
Female	209	48.95
**age**		
Under 20	26	6.10
20–29	300	70.30
30–39	89	20.80
40–49	9	2.10
Over 50	3	0.70
**education**		
High school or below	62	14.52
Associate degree	115	26.93
Bachelor’s degree	237	55.50
Master’s degree or above	13	3.04
**job**		
Management	35	8.20
Professional and technical	175	40.98
Sales, marketing, and public relations	91	21.31
Administrative and clerical	27	6.32
Production and operations	9	2.11
Customer service and support	68	15.93
Others	22	5.15
**income**		
less than CNY 3000	176	41.22
CNY 3000–5000	167	39.11
CNY 5000–10,000	57	13.35
More than CNY 10,000	27	6.32
**use experience**		
1–3 times in the last month	164	38.41
3–5 times in the last month	177	41.45
5–10 times in the last month	56	13.11
More than 10 times in the last month	30	7.03

**Table 3:** Confirmatory factor analysis and reliability.

Constructs	Indicators	Factor Loadings	CR	AVE	Cronbach’α
ATT	ATT1	0.898	0.916	0.785	0.916
	ATT2	0.885			
	ATT3	0.874			
SN	SN1	0.816	0.883	0.716	0.881
	SN2	0.883			
	SN3	0.838			
PTE	PTE1	0.796	0.888	0.664	0.887
	PTE2	0.840			
	PTE3	0.800			
	PTE4	0.823			
PCB	PCB1	0.807	0.847	0.649	0.846
	PCB2	0.823			
	PCB3	0.786			
PU	PU1	0.793	0.884	0.656	0.883
	PU2	0.830			
	PU3	0.819			
	PU4	0.797			
PEOU	PEOU1	0.842	0.883	0.655	0.881
	PEOU2	0.863			
	PEOU3	0.747			
	PEOU4	0.781			
PR	PR1	0.823	0.848	0.651	0.845
	PR2	0.860			
	PR3	0.733			
IU	IU1	0.785	0.831	0.621	0.845
	IU2	0.825			
	IU3	0.752			

CR: composite reliability; AVE: average variance extracted.

**Table 4:** Measurement model.

Fit Indices	$\chi^{2}$	df	$\chi^{2}$/df	SRMR	RMSEA	GFI	IFI	CFI	TLI
Measurement model	524.275	296.000	1.771	0.028	0.043	0.919	0.972	0.972	0.967

**Table 5:** Discriminant validity.

	IU	PR	PEOU	PU	PCB	PTE	SN	ATT
IU	**0.788**							
PR	−0.586	**0.807**						
PEOU	0.738	−0.671	**0.810**					
PU	0.704	−0.607	0.760	**0.810**				
PCB	0.625	−0.544	0.691	0.715	**0.805**			
PTE	0.599	−0.458	0.595	0.745	0.667	**0.815**		
SN	0.611	−0.567	0.699	0.759	0.665	0.736	**0.846**	
ATT	0.675	−0.496	0.755	0.712	0.569	0.619	0.725	**0.886**

The bold diagonal elements are the square roots of each AVE. Construct correlations are shown as off-diagonal.

**Table 6:** Model fit index.

Fit Indices	$\chi^{2}$	df	$\chi^{2}$/df	SRMR	RMSEA	GFI	IFI	CFI	TLI
Structural model	420.950	235.000	1.791	0.030	0.043	0.926	0.975	0.975	0.970

**Table 7:** Hypothesized relation.

Hypotheses	Paths	Unstd.	S.E.	Z	*p*	Std.	Results
H1a	ATT→PEOU	0.358	0.045	8.032	***	0.476	support
H2a	SN→PEOU	0.144	0.069	2.075	*	0.152	support
H3a	PTE→PEOU	−0.041	0.066	−0.619	0.536	−0.039	reject
H4a	PCB→PEOU	0.392	0.067	5.814	***	0.349	support
H1b	ATT→PU	0.088	0.044	1.990	*	0.127	support
H2b	SN→PU	0.149	0.060	2.471	*	0.171	support
H3b	PTE→PU	0.265	0.058	4.535	***	0.277	support
H4b	PCB→PU	0.170	0.063	2.677	**	0.164	support
H5	PEOU→PU	0.243	0.064	3.775	***	0.264	support
H6	PEOU→IU	0.460	0.069	6.687	***	0.509	support
H7	PU→IU	0.291	0.072	4.021	***	0.297	support

*: *p* < 0.05; **: *p* < 0.01; ***: *p* < 0.001.

**Table 8:** Moderating effects.

Independent Variable	Model1				Model2			
	coeff	SE	t	*p*	coeff	SE	t	*p*
constant	3.831	0.035	108.356	***	3.861	0.035	108.777	***
PEOU	0.450	0.044	10.184	***	-	-	-	-
PU	-	-	-	-	0.416	0.046	9.111	***
PR	−0.283	0.049	−5.729	***	−0.317	0.049	−6.434	***
PEOU×PR	−0.129	0.031	−4.178	***	-	-	-	-
PU×PR	-	-	-	-	−0.080	0.030	−2.673	**
	$R^{2}$ = 0.441; F = 111.026, *p* < 0.001				$R^{2}$ = 0.396; F = 92.291, *p* < 0.001			

Dependent variable is IU; ***: *p* < 0.001; **: *p* < 0.01.

## Data Availability

Data are available upon request from the corresponding author.
